# A Rare Case of Neurosyphilis Manifesting as Psychosis in an HIV-Negative Patient

**DOI:** 10.7759/cureus.40064

**Published:** 2023-06-06

**Authors:** Angela Yazigi, Majed Yazigi, Hadeel Zainah

**Affiliations:** 1 Infectious Diseases, Al-Baath University, Faculty of Medicine, Homs, SYR; 2 Infectious Diseases, Kent-County Memorial Hospital, Warwick, USA

**Keywords:** tertiary syphilis, general paresis, hiv-negative, neurosyphilis, psychiatric symptoms

## Abstract

Neurosyphilis is a rare disease now, given the availability of antibiotics to treat syphilis. Patients with neurosyphilis could present with psychiatric symptoms. We present a rare case of neurosyphilis with only psychiatric symptoms. The patient was a 49-year-old male who presented with self-neglect and was not interacting with others. Treponema antibodies were positive, and rapid plasma reagin (RPR) was 1:512 with a positive venereal disease research laboratory test (VDRL) in the cerebrospinal fluid. The patient was treated with an IV penicillin regimen for neurosyphilis and improved remarkably with a return to baseline on follow-up.

## Introduction

Syphilis cases have been increasing since 2000 [1]. The rate of primary and secondary (P&S) syphilis has been rising in men and women; it increased by 6.8% in 2019-2020 [1]. The relationship between neuroinvasion and other manifestations of syphilis and the infecting strain of Treponema pallidum is not known [2]. Neurosyphilis could manifest with different symptoms depending on the stage of syphilis and the organs involved. Early neurologic clinical manifestations, or syphilitic meningitis, are usually present within the first few months or years of infection [3]. Late neurologic manifestations (e.g., tabes dorsalis and general paresis) occur 10 to >30 years after infection [3]. Neurosyphilis could cause psychosis and be mistaken for psychiatric disorders [4]. Psychiatric manifestations could happen in 33%-86% of neurosyphilis cases [5]. We present a case of neurosyphilis with psychosis as the only clinical finding. This article was previously presented as a meeting abstract at the 2022 ACP Rhode Island Chapter Scientific Meeting on March 2, 2022.

## Case presentation

**Figure 1:**
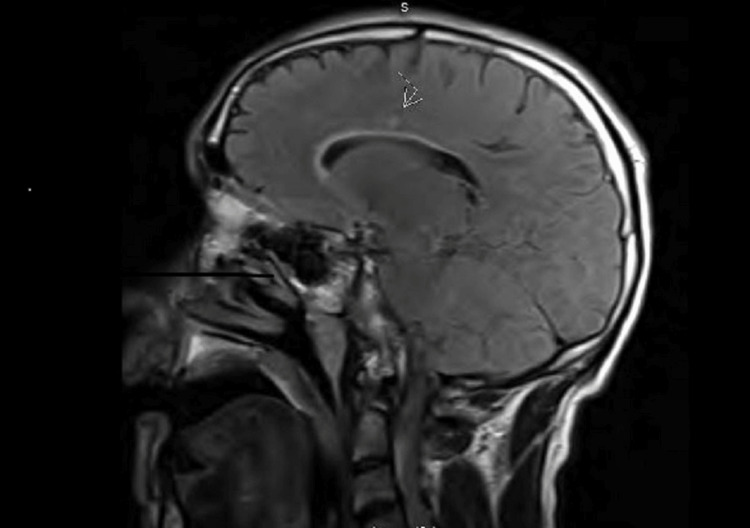
T2 FLAIR sagittal section of the brain MRI The sagittal view shows a T2/FLAIR hyperintense lesion in the periventricular white matter (arrow points to the lesion)

The patient is a 49-year-old male with a history of prior alcohol use, with previous alcohol withdrawal requiring hospitalization. The patient quit two years before his presentation. He did not have any history of psychiatric illness or sexually transmitted diseases. He presented with a two-month history of behavior change. While the patient was unable to provide a history at arrival, his family had reported visual and auditory hallucinations and paranoia. More recently, the patient had been neglecting self-care, going without eating or showering in the weeks before the presentation. He stopped talking as well. Vital signs were normal. The patient was not interactive during the exam and was initially nonverbal. He had intact strength and sluggish pupils' reactions to light. There were no penile ulcers or lymphadenopathies. A workup was started and revealed a normal complete blood count, chemistry, and hepatic enzymes. Urine toxicology screening and serum ethanol level were both negative, and a CT scan of the head did not demonstrate any acute pathology. It turned out that the patient had a previous left visual loss with persistent blurriness in his vision, with a previous brain MRI notable for multiple non-enhancing white matter lesions in the cerebrum and cerebellum. During the current admission, the patient was started on high-dose IV methylprednisolone (1 g daily x5 days) for possible autoimmune encephalitis or multiple sclerosis flares in addition to antipsychotics. Subsequent brain MRI found stable multiple non-enhancing periventricular white matter lesions (Figure 1) without associated spine lesions on spine MRI.

He was found to have a positive serum treponemal antibody and an equivocal Lyme enzyme-linked immunosorbent assay (ELISA) with a negative Lyme western blot. Serum RPR was positive at 1:512. The fourth-generation HIV antigen/antibody test was negative. Lumbar puncture was significant for 39/mcL white blood cells with 100% lymphocytes, protein of 118 mg/dL, and glucose of 68 mg/dL. VDRL in the cerebrospinal fluid (CSF) was positive. The CSF viral meningitis polymerase chain reaction (PCR) panel was negative, including cytomegalyvirus, herpes 1 and 2 viruses, enteroviruses, and some bacterial pathogens. The CSF Lyme index was unremarkable. The patient was subsequently started on IV aqueous penicillin G (4 million units every four hours). He was diagnosed with neurosyphilis and psychosis. Further CSF studies were also notable for positive oligoclonal bands, which were suspected to be due to neurosyphilis. 

The patient improved gradually throughout his admission and was seen on follow-up in the outpatient clinic with complete resolution of symptoms and an improvement in RPR to 1:64 after three months.

## Discussion

In 2020, the CDC reported 133,945 cases of all stages of syphilis in the United States [1]. The P&S syphilis rate reached its historic low in 2000-2001 but increased every year afterward, increasing by 6.8% in 2019-2020 [1]. Despite the fact that rates of P&S syphilis have increased in women in recent years, the rate in men is still higher, with homosexual men accounting for the majority (53%) of cases in men [1]. 

Neurosyphilis is a complication of untreated syphilis. Some strains of Treponema pallidum are neurotropic [2]. Central nervous system involvement could happen during any stage of syphilis, and CSF involvement could happen in early syphilis even in the absence of symptoms [3]. Early neurosyphilis may be asymptomatic or may cause meningitis, ocular syphilis, otosyphilis, or meningovascular disease and manifest in the first few months or years of infection [2]. Late syphilis could manifest as general paresis or tabes dorsalis about 10-30 years after infection but could happen earlier in immunocompromised patients. Syphilis could cause psychiatric symptoms that could range from hallucinations to schizophrenia-like symptoms [4,6]. It could even alter the course of a psychiatric illness [7]. Syphilis could also mimic depression, cognitive impairment, and delirium [8]. This large range of manifestations makes syphilis more likely to be misdiagnosed. The occurrence of psychiatric manifestations in neurosyphilis ranges from 33%-86% in the literature [5]. However, psychosis could be the only presenting symptom. In a study by Lin et al. of 169 patients who presented primarily with various psychiatric symptoms, 52 (30.77%) patients were found to have positive serum RPR and treponema pallidum particle agglutination assay (TPPA), and 44.2% had CSF pleocytosis and elevated protein, suggesting that neurosyphilis mimics many psychiatric disorders [9]. Therein, neuropsychiatric symptoms occurred in patients with general paresis [9]. MRI in neurosyphilis is usually normal or has nonspecific mild to moderate cerebral atrophy [10,11]. 

The differential diagnosis, in this case, was concerning schizophrenia, paranoia, demyelinating disorders, or a paraneoplastic disease. Despite parenchymal neurosyphilis being reported [12], it remains rare, and the lesions seen on his MRI were old. The response to syphilis treatment, despite the steroid treatment was not continued, confirmed that his psychosis was due to neurosyphilis. Furthermore, the CSF analysis and CSF VDRL were suggestive of neurosyphilis. Lin et al. reported that 16 (30.8%) of the 52 patients with neurosyphilis with psychiatric manifestations had normal protein and leukocytes in their CSF, suggesting that normal CSF analysis does not rule out syphilis with psychosis [9]. Our patient’s serum RPR was 1:512, which is consistent with the literature in neurosyphilis patients with psychiatric manifestations; RPR is always positive in those patients and could reach >1:128 [9]. The diagnosis was consistent with syphilis-induced psychosis, likely in the context of general paresis. The patient was not considered to have multiple sclerosis since there were no new lesions on the MRI. Furthermore, the presentation was atypical for primary psychosis at his age, and there was no malignancy to suspect paraneoplastic syndrome.

In HIV-negative patients, psychosis could be the primary presenting manifestation of the disease [8]. Syphilis should be suspected, especially if there is no history of psychiatric disorders, in the absence of response to antipsychotics, or late-onset psychiatric disease.

Our patient had recovered completely when he followed up after three months. Sakar et al. reported improvement six weeks after treatment [5]. Sakar et al. used 1 g of IV ceftriaxone for 14 days, while we used first-line therapy with IV penicillin.

## Conclusions

Neurosyphilis should be considered in patients with psychiatric symptoms, even in the absence of other symptoms. Physicians should keep neurosyphilis in mind, especially in late-onset psychiatric manifestations. This case confirms that syphilis could mimic any disease, including psychiatric disorders.
